# Galvanism in Extracting Teeth

**Published:** 1859-02

**Authors:** J. H. McQuillen, J. Foster Flagg, T. L. Buckingham, Jas. E. Garretson, C. N. Peirce


### GALVANISM IN EXTRACTING TEETH.

At the stated meeting of "Pennsylvania Association of Dental Surgeons," held December 7th, 1858, the Committee on Galvanism made the following report, which, on motion, was adopted:

MR. PRESIDENT: The committee appointed by the Association to investigate the merits of applying the galvanic current in the extraction of teeth, would beg leave to report, that they have attended to the duty assigned them.

Immediately after their appointment, the members of the committee were furnished with galvanic batteries, and instructed how the current should be employed, by the agent of the patentee. These bateries have been used, and the instructions followed by the committee in their investigations. Desirous of arriving at an impartial and unbiased opinion, and believing that this could only be obtained from a somewhat lengthened series of experiments, the committee have permitted several months to elapse before presenting their report.

Their experience, which has been gained in *private practice*, has varied from time to time. At one period, certain members were disposed to believe that they recognized in the application of the galvanic current, all that had been claimed for it, and all that had been said in its favor; but a later experience and more careful observation convinced them to the contrary. They have found that although in a certain proportion of cases they have had evident alleviation of suffering, and in some the assurance that the operation has been absolutely painless, yet that in the majority of instances, the pain of extraction has been unmitigated, and in some cases the application has been denounced as productive of the most excruciating suffering.

With the view of ascertaining how far galvanism was instrumental in alleviating suffering, the operator, when engaged in extracting a number of teeth for one individual, would occasionally intermit the current without apprising the patient of the fact. On being questioned relative to the sensations experienced, the answers were frequently, though not always, the same as when the current was perfectly established.

Therefore, while your committee does not feel warranted in positively condemning the use of the galvanic current, as an anæsthetic agent in the extraction of teeth, they can not, in justice to their convictions, give it that commendation which it has received from the hands of others.

Having discharged their duty, your committy would respectfully request to be dismissed from further consideration of the subject.

J. H. McQuillen,
J. Foster Flagg,
T. L. Buckingham,
Jas. E. Garretson,
C. N. Peirce.

